# Innovative Application of Standard Sand as a Functional Carrier for Nano-Silica in Cement

**DOI:** 10.3390/ma18184277

**Published:** 2025-09-12

**Authors:** Meytal Shalit, Yaniv Knop, Maya Radune, Yitzhak Mastai

**Affiliations:** 1 Department of Chemistry, The Institute of Nanotechnology, Bar-Ilan University, Ramat Gan 52900, Israel; meytal5522@gmail.com; 2 Department of Civil Engineering, Ariel University, Ariel 40700, Israel; yanivkn@ariel.ac.il (Y.K.); radune@ariel.ac.il (M.R.)

**Keywords:** nanomaterials, calcium silicate hydrate (CSH), Nano-SiO_2_, compressive strength, sand, pozzolans

## Abstract

Nano-silica (NS) is used to enhance the mechanical and durability properties of cementitious materials; however, its frequent tendency to agglomerate limits its effectiveness and uniform distribution within the cement matrix. The main goal of this study was to improve NS dispersion and therefore to improve the properties of the concrete by coating NS onto standard sand particles (sand@NS) using the Stöber method, creating a composite material that acts as a filler, nucleation site, and highly reactive pozzolanic agent. The resulting sand@NS was incorporated into cement mixtures, and its compressive strength was measured after 3, 7, and 28 days of curing. In addition, water absorption and microstructural density were also evaluated. Comparative results showed that sand@NS significantly enhanced early-age hydration and initial strength, with a 145% increase in compressive strength at 28 days compared to the reference, whereas free NS resulted in a 120% increase. The early-age strength improvement was mainly due to the increased number of nucleation centers, while later strength gains were attributed to pozzolanic activity of the immobilized NS. Additionally, sand@NS reduced water absorption and increased microstructural density, even with reduced cement content, supporting more sustainable and eco-efficient concrete production. This work shows a promising, scalable, and cost-effective strategy to maximize the performance of NS in cementitious systems and supports its broader adoption in advanced construction materials.

## 1. Introduction

In recent decades, concrete has become the most widely used construction material globally due to its durability, availability, and increased construction rate. However, its mechanical and physical properties are limited, prompting new research to enhance its performance. One of the most promising areas is the addition of nanoparticles to concrete [1,2,3]. Nanotechnology has developed rapidly, driven by continuous progress in the preparation and characterization of solid materials, which has prompted numerous benefits for construction [4,5]. Recent studies have shown that NS can significantly enhance the compressive strength of cementitious composites by filling micropores and microcracks in the cement matrix while accelerating the hydration process, resulting in a denser and more durable material [6,7]. In addition, NS improves durability by reducing permeability [3,8] and contributes to a longer service life of concrete structures [9,10,11]. These advantages make nano-silica a promising additive for sustainable construction materials.

Han et al. [12] observed the effect of NS coated with nano-titanium (NT) in concrete. They contain a structure that cures the matrix floats, which leads to stiffness of the cement combination. The NT can increase the amount of crystalline calcium hydroxide (Ca(OH)_2_) at an early age of hydration and accelerate the formation of calcium silicate hydrate (C-S-H), cement gel, which affects the strength of the concrete. Nazari et al. [13] observed concrete with four different contents of NT (0.1%, 0.5%, 1.5, and 2.0%) added by cement weight. They found that the compressive strength increased with NT content; the optimal amount, 2%, resulted in a 25% increase in compressive strength at a curing age of 28 days [9,10]. Moreover, it was reported that concrete with 5% NT particles exhibits higher compressive strength at 28 days compared to concrete without NT. The recorded increase was 11.5 MPa, a 22.1% improvement, which can be attributed to the increased amount of Ca(OH)_2_ in the crystalline structure at an early stage of hydration, accelerating the formation rate of C-S-H and improving concrete strength [6,10].

NS is one of the most effective nanomaterials used in concrete due to its high surface area, pozzolanic reactivity, and ability to enhance both mechanical strength and durability. NS can replace 20–30% of cement in the mix while improving compressive strength and reducing water permeability [3,7,8]. Its fine particles act as nucleation centers, accelerating hydration reactions and forming additional C-S-H, which densifies the matrix [14]. Both crystalline and amorphous forms of silica contribute synergistically: crystalline silica serves as a stable carrier, while amorphous silica reacts with Ca(OH)_2_ and creates C-S-H to improve packing density and reduce porosity [15,16,17,18]. This dual action leads to a more uniform microstructure, decreased permeability, and increased long-term durability [8,14,19,20].

In the construction industry, cement-based materials achieve improved properties by incorporating nanomaterials, but issues are still associated with their use. One of the main limitations of using NPs (nanoparticles) is the agglomeration between the particles due to the ultra-fine particle size, which drastically reduces the reactivity and efficiency, leading to exceptionally high surface-free energy. This agglomeration creates extensive large particle accumulation, which drastically reduces the reactivity and efficiency of the particles in the cement matrix [8,14,21,22].

Nazari et al. [13] reported that due to their large specific surface area, NT particles tend to agglomerate, limiting the improvement in concrete strength when incorporating NPs. However, Prasad Bhatta et al. [14] confirmed that the optimal inclusion of NS particles significantly enhances the properties of the cement mix. Improvements in compressive strength are observed with a concentration of 3% particles in the mixture. Justs et al. [23] employed the hydrodynamic cavitation method as a tool to disaggregate NS particles and activate their surfaces before incorporation into the cement mix. This method proved efficient and rapid, with a 9% increase in compressive strength [23].

In our previous work, we found that the fixation of NS particles on micrometer-sized polystyrene particles enhances the surface area of the NPs, making them more reactive. It was observed that the incorporation of core-shell structured polystyrene particles coated with NS into cement mixtures resulted in a 12% increase in compressive strength compared to the reference sample. In contrast, the integration of NS particles without fixation led to only a 4% increase in strength relative to the reference mixture [24]. However, the state of NP agglomeration in the resulting hydrated cement paste has not been thoroughly investigated [19,21].

This study proposes a novel method to overcome the common agglomeration problem of non-coated NS particles by fixing them onto sand particles (sand@NS). While previous research on NS in cementitious materials has mostly focused on direct dispersion or incorporating secondary carriers such as fly ash or silica fume, the present work introduces an innovative approach that transforms an otherwise inert aggregate into an active, performance-enhancing component. Although the use of sand coated with NS in cement mixtures remains largely unexplored, SiO_2_ is known to improve the mechanical properties of concrete through its pozzolanic activity by increasing packing density and accelerating cement hydration. The improved dispersion and pozzolanic reactivity of sand@NS are expected to synergistically enhance the mechanical properties and reduce the permeability of cement mixtures.

Moreover, these benefits could allow for a reduction in the total cement content, which is particularly significant given the cement industry’s contribution of about 8% to global CO_2_ emissions. This approach not only enhances concrete performance with less cement but also supports the development of more sustainable and environmentally responsible construction practices [6,14,25,26].

## 2. Materials and Methods

Portland cement Type CEM I 52.5 N (Nesher, Ramla, Israel) was used in this study (the chemical composition is provided in Supplementary Table S1). NS was prepared with an average particle size of ~187 nm. Standard sand-type certified CEN, EN 196-1 [27], was utilized. The following analytical-grade chemicals were purchased from commercial sources: tetraethyl orthosilicate (TEOS, 98%, Sigma-Aldrich, St. Louis, MO, USA), ammonium hydroxide (28%, Sigma-Aldrich, St. Louis, MO, USA) and ethanol (99%, Merck, Darmstadt, Germany). All chemicals were reagent grade and used as received without further purification. Deionized water was used throughout the investigation.

### 2.1. Synthesis of NS Particles

Silica NPs were prepared using the Stöber method [28], a widely recognized technique for controlled production of uniform silica NPs, by mixing 1.25 mL of TEOS, 15.6 mL of ethanol, 827 µL of ammonium hydroxide solution and 2.16 mL of distilled water at room temperature (RT). The synthesis reaction was carried out in a rotary shaker at RT for 18 h. After the reaction, the particle suspension was washed several times with ethanol and water, followed by centrifugation to remove unreacted materials attached to the surface of the silica particles. The NPs were characterized by SEM and XRD. SEM was used to examine the particle size, morphology, and surface texture, providing visual evidence of nanoparticle dispersion in cement pastes and coating on sand. XRD was employed to identify the crystalline phases and confirm the structural integrity of the nanosilica.

### 2.2. Synthesis of Sand@NS Particles

Composite-structured sand@NS nanospheres were synthesized via hydrolysis–condensation of TEOS in an alcoholic solution, with water and ammonia serving as catalysts. Standard silica sand (EN 196-1 [27]) was employed as a uniform substrate for NS deposition, ensuring consistent coating. The resulting homogeneous NS layer on the sand surface enhanced surface area, improved dispersion, and increased pozzolanic reactivity, thereby improving its suitability for high-performance concrete applications [28,29,30].

The sand@NS was coated by mixing 50 g of sand particles with 60 mL of ethanol to obtain a uniform suspension, and adding 105 mL of water, 22.75 mL of ammonium hydroxide, and 52.5 mL of TEOS to the suspension. The coating reaction was carried out at RT in a rotary shaker overnight. The composites were washed with ethanol and water and characterized by SEM and XRD [20,31].

### 2.3. Preparation of Cement Mixtures

Cement mixtures were prepared to evaluate the effect and activity of NS with and without fixation on the properties of the mixtures. Free-floating NS particles and sand@NS were added to the cement, where the NS was fixed onto a sand template. The water-to-cement ratio was constant throughout the experiments (*w*/*c* = 0.5), with each mixture prepared using 200 g of cement and 100 mL of water. Each mix was prepared as per the compositions presented in Table 1. The sample preparation and testing procedures were carried out in accordance with the EN 196 standard [27].

The mixing procedure was as follows: first, the cement was mixed with each of the additives (NS or Sand@NS) for 3 min. Then, water was added to the dry mix, and the paste was mixed for another 5 min. The fresh paste was cast into 25 × 25 × 25 mm molds. Finally, after 24 h, all specimens were cured in water at 20 ± 3 °C. Compressive strength was tested after 3, 7 and 28 days.

### 2.4. Characterization of Materials and Cement Pastes

The morphologies of sand, NS, sand@NS particles, and cement pastes were examined by SEM (Quanta FEG 250, FEI Company, Hillsboro, OR, USA). The morphology of NS particles was also analyzed by TEM (Tecnai G2, FEI Company, Hillsboro, OR, USA). Elemental analysis of sand was conducted using EDS. The influence of the cement replacements on the properties of the cement paste was investigated by XRD (Rigaku SmartLab (Tokyo, Japan) 3 kW diffractometer with 0.154 nm CuKα radiation source). The water absorption properties of cement samples were evaluated by immersing the specimens in water for 24 h at a temperature of 21 ± 3 °C. During this period, the pores in the cement mixtures absorbed water. After 24 h, the samples were weighed, and the difference in mass before and after soaking was obtained to ensure the end of the absorption process, in accordance with IS 26 Part 5.1. The following equation was included to calculate water absorption (%), with explicit definition of the parameters: Saturated Surface Dry Weight (Wssd) of the sample (g) and Oven Dry Weight (Wod) of the sample (g).

$$Absorption\;capacity,\;\%=\frac{\left(\mathrm{Wssd}\;\mathrm{saturated}\;\mathrm{surface}\;\mathrm{dry},g)-(\mathrm{Wod}\;\mathrm{oven}\;\mathrm{dry},g\right)}{\left(\mathrm{Wod}\;\mathrm{oven}\;\mathrm{dry},g\right)}\times 100$$

The compressive strength of the cement pastes was measured at 3, 7, and 28 days using a universal testing machine, in which specimens were placed between two plates and subjected to a progressively increasing load until failure following the procedure specified in EN 196 [27]. Three replicates were tested for each sample (n = 3).

## 3. Results and Discussion

### 3.1. Microstructure, Chemical and Morphological Characterization of Sand and Sand@NS

**Figure 1:**
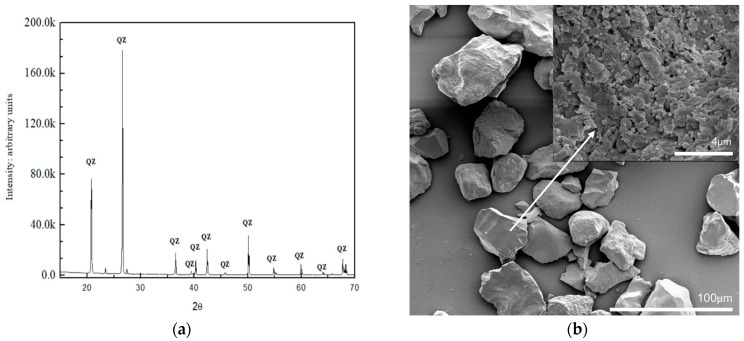
(**a**) XRD pattern; QZ: Quartz. and (**b**) SEM image of sand.

**Figure 2:**
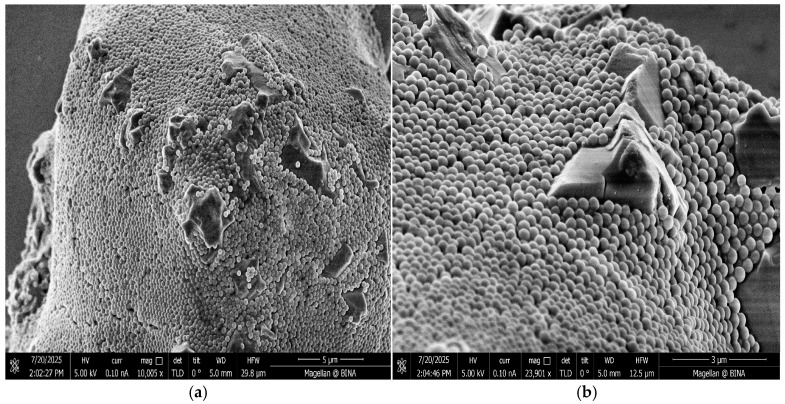
SEM images of sand@NS particles at 10 k (**a**) and 24 k (**b**) magnification.

Detailed characterization of the NS particles, including SEM and TEM images, particle size distribution, and XRD measurement, is provided in the Supplementary Material (Figures S1–S4). Standard sand is a well-defined reference material commonly used in cement testing, known for its consistent particle size and composition. In this study, standard sand served as a substrate for the coating of silica NPs, resulting in a composite material with enhanced surface area and reactivity. The uniformity in particle size and high purity of this sand make it an ideal carrier for nanomaterials, promoting improved dispersion and enabling potential applications in advanced cementitious systems. The chemical composition of the sand, determined using energy dispersive X-ray spectroscopy (EDAX), is provided in the Supplementary Material (Figure S5). The SEM image and XRD pattern of sand are shown in Figure 1, and the SEM images of sand@NS are shown in Figure 2.

Figure 1 presents the XRD pattern and SEM images of the control quartz sand, illustrating both its crystalline structure and particle morphology.

XRD reveals several sharp crystalline peaks in the angular range of 10° to 70°. The observed sharp crystalline peaks are attributed, among other things, to the crystalline phases of quartz SiO_2_ (ICSD 201354). Paul et al. [32] found that the main peak observed at 2θ = 26.67° is primarily associated with quartz (SiO_2_), which is the dominant mineral in the control sand. SEM images show quartz sand particles at two magnifications. The low-magnification image (background) shows a broad size distribution and highly irregular, angular morphology, typical of crushed quartz. The particle size ranges from 10 to 20 µm. The zoomed-in inset highlights the detailed surface texture of a selected sand grain, further illustrating the rough and angular nature of the particles.

To examine the morphology of the silica-coated sand particles, SEM analysis was performed, as shown in Figure 2.

The sand particles display a nearly complete and uniform NS coating, 187 nm in size, forming a continuous layer over the entire surface with minimal exposed areas. This indicates high surface coverage, effective nanoparticle immobilization, and good dispersion of the NS. As shown in Figure 2, the NS particles appear as small, bright white dots that densely populate the sand surface, illustrating the successful formation of the silica coating. Additionally, the strong adhesion between the NS and sand particles can likely be attributed to the high surface area of the NPs, which provides numerous contact points and enables stronger bonding through chemical interactions involving silanol groups (Si-OH) [33,34]. These hydroxyl groups form hydrogen bonds with one another, creating strong chemical interactions that reinforce the adhesion between the NPs and the sand. Silanol groups can be detected by FTIR, where they are characterized by a distinctive stretching band around 960 cm^−1^, confirming their presence on the nanoparticle surface [35]. The bonds formed via silanol groups contribute to a stable and durable interface, ensuring that the NS coating remains securely attached to the sand particles, making it suitable for pozzolanic reactivity in cementitious materials. Free NS particles tend to agglomerate, forming irregular clusters, whereas according to SEM images (Figure 2), sand@NS exhibits a uniform coating. This uniform distribution not only enhances the availability of nucleation sites for C-S-H gel formation during cement hydration but also improves particle dispersion. Overall, the SEM results demonstrate that sand@NS exhibits more uniform surface coverage than free NS particles.

### 3.2. Characterization of Cement Mixtures

**Figure 3:**
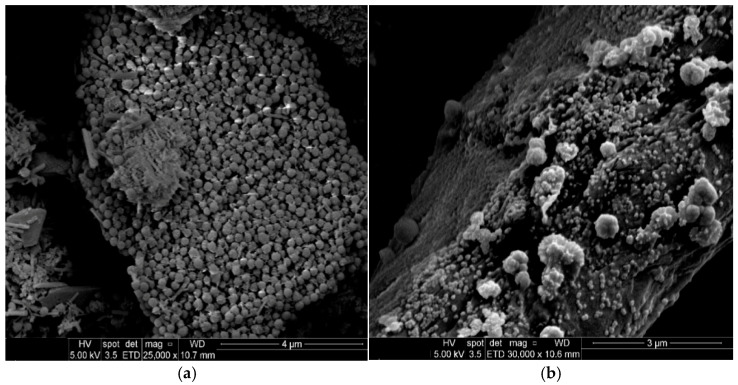
SEM images of (**a**) NS particles and (**b**) sand@NS added to cement at 3 days.

Two main reactions take place in the cement pastes: (i) hydration of the cement with water, which occurs rapidly at early ages, and (ii) the pozzolanic reaction of the NS with Ca(OH)_2_, which occurs in parallel but proceeds more slowly and continues at later curing ages. To investigate the pozzolanic effect, cement mixtures were prepared with silica NPs in two forms: uncoated NS and sand@NS (with or without fixation). The pozzolanic effect arises from the reaction between silica NPs and Ca(OH)_2_ produced during cement hydration, forming additional C-S-H gel. This reaction improves the microstructure, strength, and durability of the cementitious material. Examining both NS and sand@NS allows assessment of how the nanoparticle coating and fixation on sand influences reactivity and contributes to the performance of the cement mixtures. SEM images of NS and sand@NS particles incorporated into a cement mixture after 3 days are presented in Figure 3.

The limitations on the use of NPs can be observed, as NS particles tend to agglomerate easily. This aggregation affects their dispersion and interaction with the surrounding medium, ultimately leading to a decrease in reactivity. Yang et al. [22] found that the pozzolanic activity of colloidal NS enhances cement hydration, but its stability in cement paste is affected by the disruption of electrostatic repulsion caused by calcium ions. This disruption occurs due to the high electronegativity of NS, and when it comes into contact with cementitious materials, the electrostatic repulsion between NS particles diminishes due to their high ionic strength and the presence of multivalent cations in the paste. Consequently, this leads to the destabilization of NS and the formation of NS aggregates [22].

Figure 3b presents a SEM image of sand particles coated with silica NPs. The cement gel formed during the hydration process with sand@NS particles after 3 days can be observed. In this sample, the NS particles exhibit increased reactivity, as can be seen in the following figures. Due to their increased surface area, which results from their attachment to the sand particles serving as a template, the C-S-H gel phase plays a key role in the structure of fully hydrated Portland cement paste. This phase is essential in determining the properties of materials and is primarily responsible for the strength development of the hardened cement paste [22,36,37].

XRD patterns provide important insights into the crystalline phase composition of cement pastes by identifying characteristic phases. In the obtained diffractograms, the peak at 2θ ≈ 18° corresponds to Ca(OH)_2_, a key hydration product of cement. Other relevant peaks are associated with the primary clinker phases, such as alite (Ca_3_SiO_5_) and belite (Ca_2_SiO_4_), which contribute to cement hydration and strength development. The intensity of these peaks is generally proportional to the quantity of the corresponding crystalline phase [38,39]. However, in the later stages of cement hydration, pozzolanic activity occurs, leading to increased formation of the cementitious gel. As this gel is amorphous, it does not produce distinct diffraction peaks in XRD measurements, even though it represents a significant portion of the material. Consequently, the reduction in Ca(OH)_2_, alite, and belite peak intensity at advanced curing ages indicates their consumption in the pozzolanic reaction, supporting the formation of an amorphous cementitious matrix.

**Figure 4:**
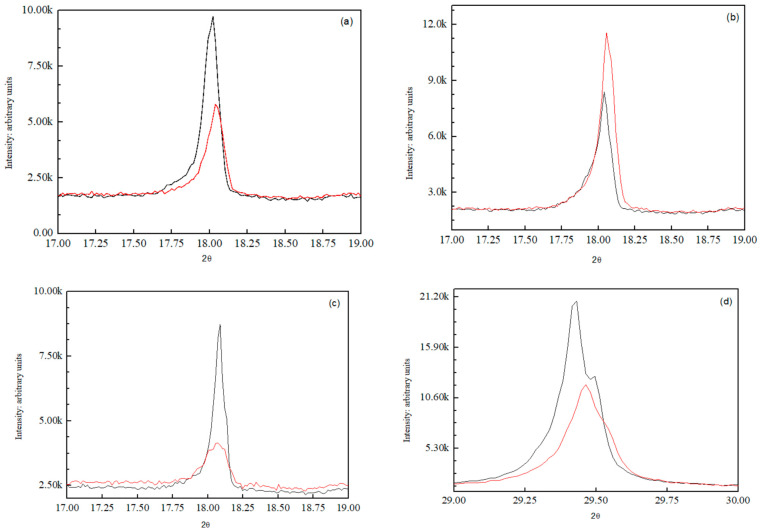
XRD patterns of cement pastes after 3 days (**a**), 7 days (**b**), and 90 days (**c**,**d**). Black lines correspond to NS and red lines to sand@NS.

Figure 4 shows the X-ray diffraction patterns of the cement pastes at different curing times: 3 days (a), 7 days (b), and 90 days (c and d). Figure 4a shows that the amount of Ca(OH)_2_ formed during the hydration process after 3 days is higher in the mixture containing NS compared to the sand@NS sample. As the concentration of NS particles increases, so does the production of Ca(OH)_2_, as NS acts as nucleation centers that accelerate hydration. Consequently, a greater amount of NS leads to more nucleation centers, resulting in a corresponding increase in the amount of Ca(OH)_2_. Figure 4b illustrates the significant pozzolanic activity observed after 7 days in the cement mixture with sand@NS, where the highest peak is attributed to it. After 90 days, Figure 4c achieves the opposite result: the more sand@NS, the lower the amount of Ca(OH)_2_. Within this time frame, the generated Ca(OH)_2_ reacts with the NS, forming a non-crystalline cement gel according to the pozzolanic reaction: NS + Ca(OH)_2_ → C-S-H [40]. Therefore, a low amount of Ca(OH)_2_ indicates a higher pozzolanic capability. Given the pozzolanic activity of NS, its coating on the surface of sand particles can react with Ca(OH)_2_, decreasing the intensity of the peak as more Ca(OH)_2_ is used, leading to an increase in the formation of cement gel. The peak in Figure 4d corresponds to the minerals alite (ICSD 162744) and belite (ICSD 245075), which are formed as a result of nucleation centers during the hydration process. This figure also shows the inverse trend, indicating the formation of cement gel, as evidenced by the decreasing intensity of the sand@NS peak compared to the mixture with NS.

#### Compressive Strength, Absorption, and Specific Weight of Cement Pastes

**Figure 5:**
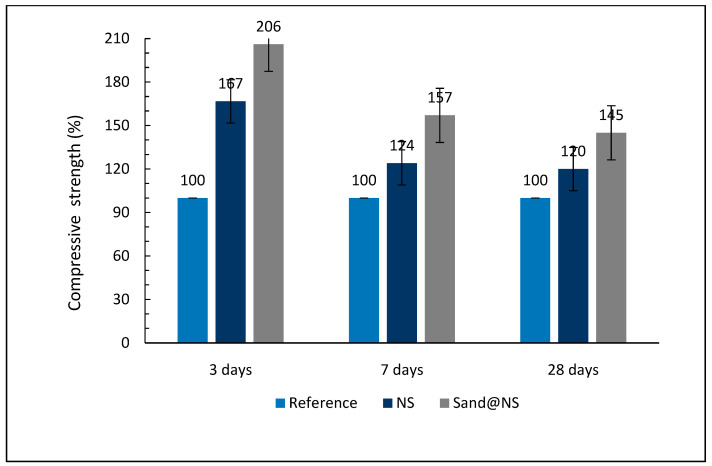
Compressive strength of samples incorporated into cement after 3, 7 and 28 days of curing.

The compressive strength of the cement samples was measured after 3, 7, and 28 days of curing, and the results are presented in Figure 5. Concrete’s strength is one of the most important properties that determines its durability and structural performance. This strength develops over time through the hydration reaction in which cement particles react with water to form a solid, hardened matrix. Initially, this process leads to the formation of Ca(OH)_2_ and C-S-H gel, which contributes to the mechanical properties of concrete [41,42].

Adding pozzolanic materials, such as NS, enhances this process by reacting with Ca(OH)_2_ to generate additional C-S-H gel, improving both strength and durability. However, strength development is not uniform over time. At early stages, nucleation effects dominate, accelerating the initial hydration process. In later stages, pozzolanic activity plays a more significant role, leading to the formation of a denser and harder microstructure that further enhances long-term strength [19,21,38].

The compressive strength development was measured 3, 7, and 28 days after casting for three samples: reference, NS, and Sand@NS.

After 3 days, a significant increase in strength was observed in the Sand@NS sample compared to the reference and NS. This increase in early-age strength is attributed to the greater number of nucleation centers, promoting initial cement hydration. After 7 days, the strength of Sand@NS remained significantly higher than that of the NS sample. This indicates that the increased strength results from pozzolanic activity, which is also evident after 28 days despite the decreased amount of cement (Table 1). The strength of Sand@NS (145%) remained the highest; however, the smaller difference with NS (120%) suggests that pozzolanic activity plays a more dominant role in later stages of hardening due to the slow reaction rate of the pozzolanic reaction. The nucleation effect in Sand@NS was particularly dominant during the early stages (3 days), as it resulted in an increased number of nucleation sites for cement gel formation. Over time, pozzolanic activity, which requires Ca(OH)_2_ for cement gel formation, became more pronounced and contributed to continued strength development in both NS and Sand@NS samples.

In this study, incorporating NPs into the cement blend led to a reduction in Ca(OH)_2_ content in the sample containing NS. This reduction is attributed to the pozzolanic activity of NS, which formed a coating on the surface of Sand@NS. During the pozzolanic reaction, NS particles reacted with Ca(OH)_2_ to form C-S-H, thereby enhancing the strength and durability of the cement matrix. In contrast, the NS sample without coating did not exhibit significant pozzolanic activity, as evidenced by the absence of a substantial decrease in Ca(OH)_2_ content [16,18,36,40,42,43,44].

The strength measurements indicate that despite the reduction in cement content, NS particles fixed onto sand exhibited greater strength compared to free-floating particles, demonstrating improved properties of the cement mixture due to the enhanced pozzolanic effect of NS when immobilized onto a template, the active component in cement. Greater strengths were achieved with the addition of NS (as shown in Figure 5) [45].

**Figure 6:**
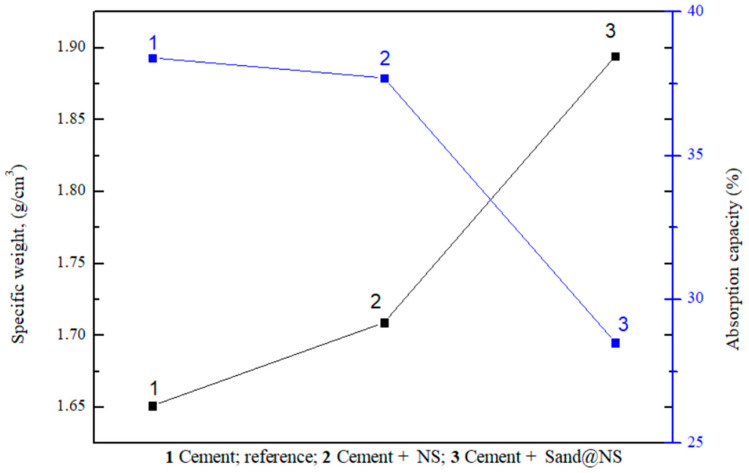
Absorption and specific weight measurements of cement pastes.

The water absorption and specific weight of the cement pastes were measured, and are presented in Figure 6. Water absorption is another important property of concrete, influencing its durability, overall performance, and sustainability. The amount of adsorbed water can impact the cement strength, while lower water absorption is generally associated with reduced porosity, which contributes to enhanced strength and durability [46]. The incorporation of NS and sand@NS into cement mixtures affects both the specific weight and water absorption of the concrete. The specific weight reflects the density of the mixture, which is closely related to its porosity and water absorption characteristics. Concrete with higher density has lower porosity, thereby reducing its ability to absorb water.

The effects of adding NS and sand@NS on the cement mixtures, specific weight, and absorption are shown in Figure 6. Adding NS, especially in the form of sand@NS, increased the cement paste density while decreasing water absorption. With a specific weight of 1.9 g/cm^3^, the cement + Sand@NS sample demonstrated a notable increase in density in comparison to both cement + NS (1.7 g/cm^3^) and the reference sample (1.6 g/cm^3^), suggesting that a denser mixture with better sealing and hardened concrete performances was obtained. Kumar et al. [46] showed that water absorption has an inverse relationship with compressive strength, with higher water absorption corresponding to lower compressive strength and vice versa across all concrete sets.

The increased density (lower water absorption) of the samples with NPs and especially with sand@NS indicates that a higher density and lower porosity of the final cement samples was obtained. The improved properties of the hardened cement are attributed to two main factors: the first is the increased packing density due to nano-scale particles with lower agglomeration, and the second is the increased hydration and pozzolanic reaction rate and degree. The latter is due to two different chemical reactions, cement hydration and the pozzolanic reaction, which create a cement gel that increases the compressive strength of the concrete [42,46,47].

## 4. Conclusions

This work presents an innovative and practical method to overcome the agglomeration of particles due to intermolecular forces. This phenomenon is considered one of the main obstacles to the use of NPs in concrete. By immobilizing NS on the surface of standard sand particles (sand@NS), better dispersion, enhanced hydration, and improved pozzolanic performance were achieved without increasing material complexity or cost. Notably, this method led to a significant improvement in the hardening properties of the cement by increasing compressive strength and reducing permeability, even with lower cement content, offering both mechanical and environmental advantages. This template-assisted approach, which transforms a passive component (sand) into an active functional carrier for nanomaterials, provides a new pathway for integrating NPs into cementitious materials effectively and sustainably. Further research into the scalability and optimization of sand@NS in various construction contexts is warranted.

## Figures and Tables

**Table 1:** Mix design of cement samples.

(wt.%)	Sample 1	Sample 2	Sample 3
Cement	66.67	65.36	55.56
Water	33.33	32.68	27.78
NS	0	1.96	0
Sand@NS	0	0	16.66

All values are given in weight percent (wt.%) relative to cement pastes.

## Data Availability

The original contributions presented in this study are included in the article/Supplementary Materials. Further inquiries can be directed to the corresponding author.

## Supplementary Materials

The following supporting information can be downloaded at: https://www.mdpi.com/article/10.3390/ma18184277/s1, Table S1: Chemical composition of cement (wt%); Figure S1: TEM image of monodisperse silica nanoparticles; Figure S2: SEM image of silica nanoparticles; Figure S3: XRD pattern of silica nanoparticles Figure S4: DLS size distribution graph of NS; Figure S5: EDX spectrum of sand.
