# Supplementary material for: Innovative Application of Standard Sand as a Functional Carrier for Nano-Silica in Cement
Source: Materials (Basel). 2025 Sep 12;18(18):4277. doi: 10.3390/ma18184277 (PMC12471318; doi:10.3390/ma18184277)
Supplement: Supplementary file 1 [file materials-18-04277-s001.zip › materials-3845377-supplementary.pdf]

Supplementary Materials

Innovative Application of Standard Sand as a Functional Carrier for Nano-Silica in Cement

Shalit M, Knop Y, Radune M, Mastai Y*

* Corresponding author. Email: mastai@biu.ac.il

Composition	Quantity (wt.%)
CaO	63.47
SiO_2	18.52
Al_2O_3	5.3
Fe_2O_3	3.45
MgO	1.00
TiO_2	0.36
K_2O	0.43
Na_2O	0.22
P_2O_5	0.31
Mn_2O_3	0.04

Table S1: Chemical composition of cement (wt%). Analysis performed by ICP-OES

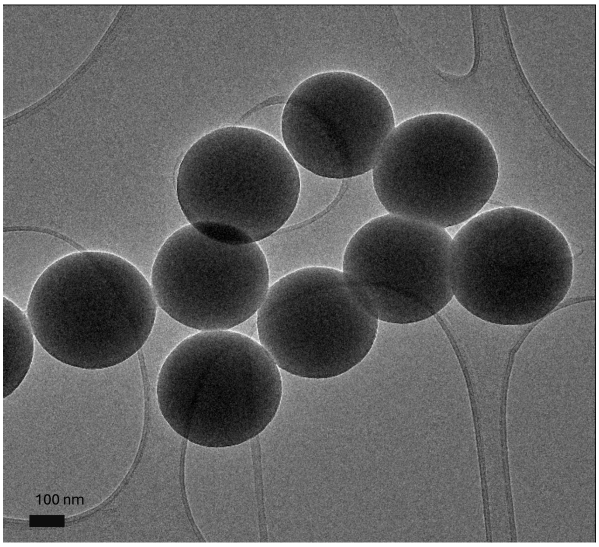

Figure S1: TEM image of monodisperse silica nanoparticles. The image shows high contrast, where the dark areas represent the dense silica cores and the lighter regions indicate the surrounding medium.

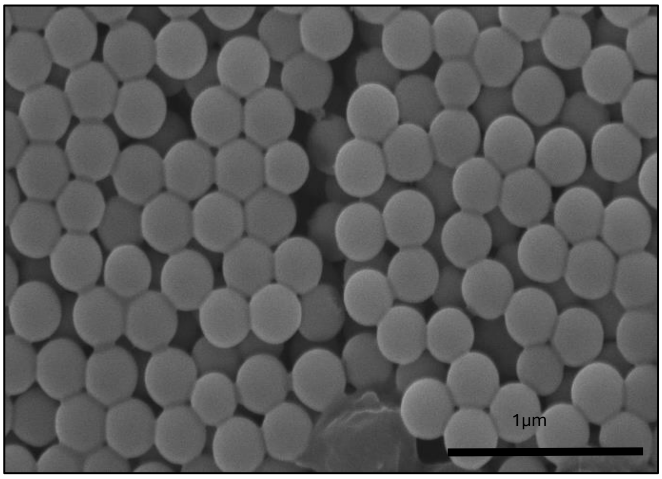

Figure S2: SEM image of silica nanoparticles showing uniform spherical morphology, with slight agglomeration observed.

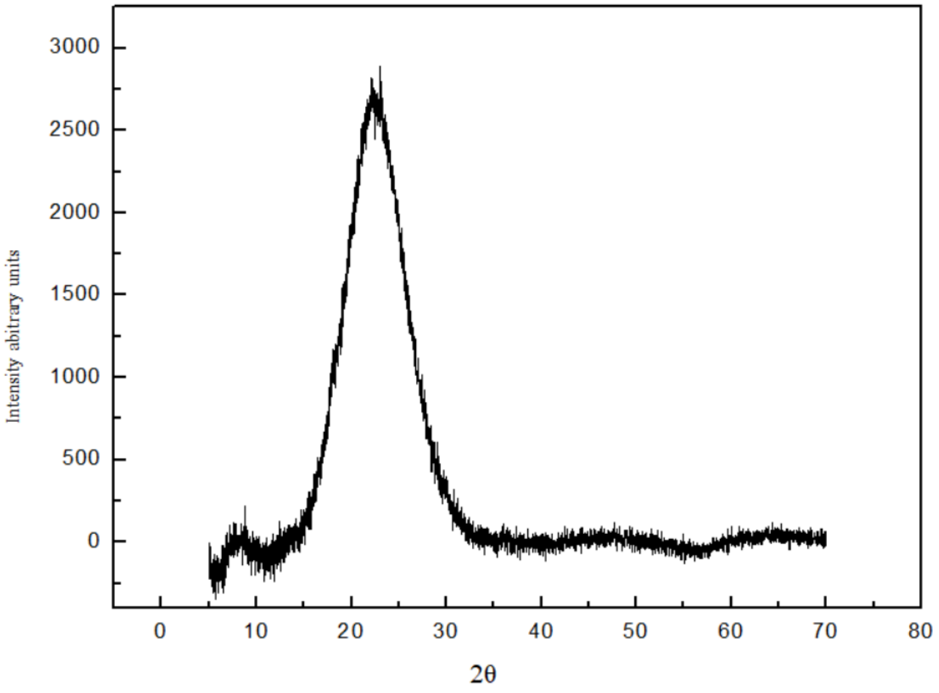

Figure S3: XRD pattern of silica nanoparticles indicating their amorphous nature. The broad peak around 22° (2θ) confirms the absence of long-range order, which is characteristic of amorphous silica.

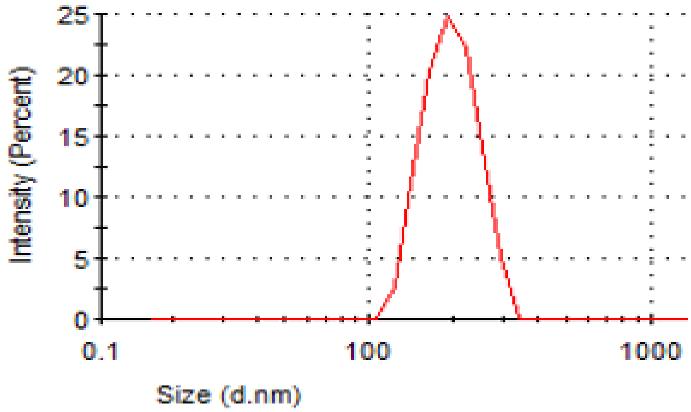

Figure S4: DLS size distribution graph of NS. The shows a sharp peak in the nanometer range, with an average hydrodynamic diameter of 187 nm ± 14.2 nm

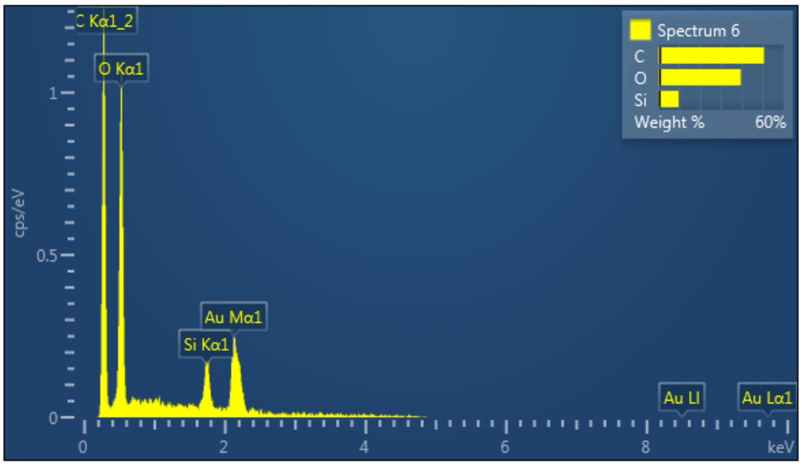

Figure S5: The EDX spectrum of the sand sample confirms the presence of silicon (Si) and oxygen (O) as the main elements. The dominant peaks of Si and O are consistent with a silica-based composition. The gold (Au) peaks originate from the conductive coating applied for SEM analysis. The carbon (C) peak is attributed to using carbon tape for sample mounting.
